# Letter from Dr. De Carro to the Editors of the Bibliothèque Britannique, on the Guinea Worm, and the Sting of the Scorpions of India
*From Bibliothéque Britannique, vol. xxx.


**Published:** 1807-02

**Authors:** 


### *Letter from Dr.* DeCarro *to the Editors of the Bibliothèque Britannique, on the Guinea Worm, and the Sting of the Scorpions of India.**

Gentlemen, *Vienna, Sept.* 24, 1805.

When I last wrote to you on that subject of the celebrated quadruped the shawl goat of India†, I think I informed you that I intended to lay my philosophical friends in Asia under contribution, by requesting communications from them of any thing remarkable in medicine, surgery, or the materia medica. The most zealous and most regular of my correspondents is Dr. Milne, at present residing at Goa, and from him I have received some interesting details upon two of the diseases of India, as communicated by the Rev. Mr. Dubois, the missionary, to Dr. Anderson, physician-general to the government of Madras; and Dr. Anderson's answer; both of which are subjoined.

These observations upon the Guinea worm *(dracunculus)* will be so much the more interesting to your readers, as a short time ago you gave an account of the work of Mr. M'Gregor upon the diseases of the expedition to Egypt‡. The following details cannot fail to arrest the attention of the public, when we consider that they describe the efficacy of a remedy, which seemed to be completely unknown to Mr. M'Gregor and such medical men as have written on the subject:

### *Letter of the Rev. Mr. Dubois to Dr. Anderson, Physician-general.*

Dear Sir,

The principal object of this letter is to communicate to you two remedies, the efficacy of which I have remarked in a great number of instances, in two diseases very common in this district and in some others: I allude to the disease known among Europeans by the name of the *Guinea worm,* among the Indians by the name of *naramboo* or *nurapoo-chalandy*; and to the pain occasioned by the bite of a scorpion.

These two simple remedies, both of them, I suppose, equally innocent, were communicated to me by a Hindoo physician, who has administered them, with very great suc-

 cess,

* From *Bibliothéque Britannique,* vol. xxx.

† See Philosophical Magazine, vol. xxiv. p. 97.

‡ See Philosophical Magazine, vol. xxii. p. 350.

cess, to a great number of diseased persons who have daily implored his assistance. As this Hindoo was under obligations to me, I requested him to impart these remedies; which he immediately did: and as he enjoined no secrecy upon me, I do not think I shall abuse his confidence in making his communication public.

From the moment I first knew these remedies, I have also had many opportunities of experiencing the efficacy of the one against the Guinea worm in particular, and almost always with success, except in two or three instances, where the persons who had taken the remedy the first time refused to take it a second time; which is often necessary in obstinate cases. But however inveterate the case was, the remedy, upon the second application, forced the departure of the worm, if already formed; or hindered it from forming at all, when taken at the commencement of the disease.

As to the remedy against the bite of scorpions, I have had but few opportunities of trying it, having only administered it three times with equal success. I administered it one hour after the bite, at the very moment the pain was greatest: in half an hour after the pain was greatly diminished, and in an hour it totally ceased.

It is generally known, that the reason why the disease called the Guinea worm is painful and troublesome is because it particularly attacks the legs, often rendering them entirely motionless for many months.

When this disease appears in this and some other districts of the Carnatic and of Madura, it becomes sometimes epidemic to such a degree, that I have seen villages the one-half of the inhabitants of which were attacked at the same time.

Although it appears at all seasons, yet it is much more general in the months of December, January, and February; it then rages in several districts at once: at other seasons fewer persons are affected with it.

The symptoms of this malady are almost always the same. A disagreeable sensation is felt for a few days, accompanied by head-ach, pain in the stomach, and nausea; two or three days before the pain is fixed in the place in which the worm appears, an eruption of small pimples takes place, accompanied with a very disagreeable itching; this itching becomes more violent at the place where the worm is about to force a passage, until the pain is entirely fixed to one place. The part then swells, and inflammation soon follows.

In

In this case, the worm often comes away with the pus, or when the suppuration is upon the point of ceasing; sometimes the place where it is lodged swells like a bladder full of limpid matter, and sometimes only a simple induration is seen, without inflammation.

The period necessary for the discharge of the worm varies much; sometimes it happens at the end of ten days, and sometimes not for two or three months, and during all this time the limb continues in a state of inflammation and suppuration.

Sometimes it comes out all at once, if means are taken to facilitate it, and then it is often alive. I have frequently seen these insects move about for several minutes after coming out of the leg. In general, however, it comes out by little and little. About an inch of it comes out in a day, and it is carefully rolled round a straw, or something else, to hinder it from returning.

If drawn out roughly it breaks, and the piece which remains occasions swelling and inflammation, accompanied by acute pain and followed by suppuration. In this case, the disease becomes very obstinate and very painful.

However severe this disease is, it is never followed by gangrene; it sometimes happens, however, that the principal nerves are so injured that the limb becomes short or deformed.

These worms are of different sizes; some of them I saw were a foot and a half long, although they are commonly shorter; but in general their size and shape may be compared to the small string of a violin.

It is at the extremities, particularly the legs, that they appear, and sometimes in the arms. Some Hindoos assured me that they had seen them in the nose, the ears, the eye-brows, &c.; but I have never seen them except in the legs and arms.

I do not feel myself capable to divine the origin and causes of this singular disease. The Hindoos, accustomed to attribute the most of their diseases to the bad qualities of the water they use, generally believe that element to be the cause; pretending the worms are there engendered, and, being swallowed, that they increase in the human body until they find an outlet. According to this system, it would be very difficult to explain how these insects could pass through the organs of deglutition and digestion in a state of life. On the other hand, admitting that water has no part in their formation, it will be difficult to explain it. 1st, How does it happen that the inhabitants of a village who who drink the water from the same well are attacked with this disease, while others, at the distance of half a mile, are not attacked? 2d, How does it happen that those who live at the distance of a mile from a river, and who are obliged to drink well water, are almost regularly every year attacked with the above?

The climate also may have some effect upon this malady. It really attacks those who live near the sea-shore. I never saw it in any part of the Mysore, and it is even not known by name beyond the Ghauts; while it rages continually in the Carnatic, and other districts within the Ghauts, the neighbourhood of rivers excepted, as I have already remarked.

But whatever is the cause and origin of this disease, I shall be happy if the remedy I propose be a specific against it. The following example will at least prove its efficacy.

A long time before this, remedy was known to me, I was told that the Bramins were never, or at least very rarely, attacked with the above disease, although living in villages the inhabitants of which suffered constantly from it every year. I paid no attention to this observation, regarding it as one of those assertions without foundation so common among the Hindoos, who consider this exemption as belonging to the sacred character of this class of men. Reflecting afterwards upon this assertion, after knowing the remedy, I explained it by recurring to the quantity of assafœtida which the Bramins daily and constantly use in their victuals as a seasoning; and this also is the principal ingredient of the remedy.

The remedy employed against the bite of a scorpion may be applied with the same success against the sting of winged insects, according to the following recipe;—The shrub, the root of which is a specific in this case, is called by the Hindoos *kaletchy-chaddy*. Being ignorant of the name given to this shrub by European botanists, I send you two specimens of the fruit, which are called *kaletchy-kabye*, in order that you may ascertain the shrub which produced them. They are found of different hues; but, as there are various species of them, I have to caution you to choose such as have their fruit of a white colour, because the root of the other kinds has not the same virtue.

### *Remedy against the Bite of Scorpions.*

Take, at the new moon, the root of the shrub called kaletchy-chaddy, of the kind which produces white fruit; cut them into pieces of three or four inches long, and allow them to dry in the shade.

When

When a person has been bitten by scorpions, cut off a piece of root as large as a bean; let the patient place it in his mouth, pressing it gently with his teeth; he must swallow his saliva, taking a new piece of the root every ten minutes. At the same time pulverize a small quantity of the root, upon which throw some drops of water; and, after having made a paste, apply it to the wound.

*Remedy against the Guinea Worm.*

1st, Take of good assafœtida, called by the Tamuls *peroongahyam*, as much as will weigh seven *fanams* of gold (about three-fourths of a *ragoda).*

2d, The fruit, well known in all the gardens of India, called by the Tamuls *katricahe*, and by the Portuguese *beringelle.*

3d, Oil of Sesamum, called by the Tamuls *nalla-yennie*, in sufficient quantity to fry the *katricahe* or *beringelle.* Bruise the assafœtida, and after having divided the beringelle into three equal parts with a knife, insert one-third of the assafœtida in each portion of the fruit: after having tied them with a thread, fry the beringelle in the oil of sesamum in a convenient vessel; let the patient take one portion of the remedy upon going to bed, another next morning, and the third upon going to bed the second night; rub the part of the body where the worm is lodged with the oil which fried the fruit containing the assafœtida, for three days, and three times a-day.

If this remedy is resorted to at the commencement of the malady, it will arrest its progress and hinder the formation of the worm; if made use of after the worm is formed, it will soon bring it away; and in every case the pain ceases in three or four days, unless the disease is very obstinate, when the remedy must be repeated, and it never fails on the second application.

*Dr. Anderson's Answer to the Rev. Mr. Dubois.*

DEAR SIR,

I have read your letter with great pleasure and attention, because I do not know that any thing similar has been published in the English language; and, in fact, this Guinea worm, or *dracunculus*, which you have described so exactly, is one of the most dreadful maladies to which the inhabitants of this great peninsula are subject.

What you have said upon the Hindoostanee names of these remedies, &c. is of real importance to Europeans, as enabling them to procure them from the natives when they are in want of them.

As

As the knowledge of these Tamul names, however, will be only useful in India, for the instruction of the naturalists of Europe, I have to observe, that the *kalechy*, which you recommend as a remedy against the bite of winged insects, is, according to Linnæus, of the order of *Lomentaceæ* and of the genus *Guilandina*. Botanists have distinguished two species, the *bonduc* and the *bonducella*. They have made no observation upon the colour of the seeds; but I saw a lady of Bengal, on her way to Europe, who gave her child, when attacked by an intermittent fever, these seeds of a clear brown colour, in preference to Peruvian bark.

The reason you assign why the Bramins are less subject to the Guinea worm than the other natives, led you to a very natural conclusion. I must say, however, that I have remarked the same difference in the susceptibility of our officers and that of the common soldiers, having often thirty of the latter under my care at once, and not one of the former. Not observing any difference in their food which might lead me to the same conclusion with yourself, I thought these soldiers contracted the above malady by sleeping on brick pavements, as was once the custom, and that the remedy really was to make them lie upon wooden benches, which are now erected in all our barracks and guardhouses.

We know well that the *ichneumon* fly lodges its eggs in the silk-worm, wherever it can find a corner convenient enough for its future subsistence; and we see worms every day even in the kernels of fruits, which had been deposited as an egg, without the least exterior sign; we cannot allege, with propriety, that worms enter into the bodies of animals by the organs of deglutition or digestion, when we see them in the eyes of horses and the livers of sheep.

I am inclined to believe that the Guinea worm is introduced into the human body in the same way as various other insects which deposit their progeny there, or in the state of an egg it is sucked up from the humid earth by means of the absorbents of the skin. Whatever it may be, it is our duty to do our endeavours to destroy it; and, as assafœtida is known over all the world, recourse may be had to it both as a preservative and a cure.

In my whole practice I never found any thing so efficacious as cataplasms made of *cattale* or *aloe littoralis*; a remedy which was communicated to me by a Hindoo. The saponaceous quality which it appears to possess softens the teguments when strongly inflamed, diminishes the tendency to to gangrene, and favours the coming out of the worm; which I have seen sometimes lodged in the pectoral muscles as well as in the inferior extremities.

If you reflect, that insects spread themselves in clouds over districts where we never saw them before, you will find it very difficult to ascertain the local situation of certain species, such as those known by the name of the Guinea worm, while our own vessels alone frequent the mouths of some rivers in Africa, whence we first learned the knowledge of them. Rajamundry, which is situated on the banks of the Codavery, the largest river on the Malabar coast, is very subject to it: we know also that it is very common at Trichinopoly, upon the river Cavery; and even at Bombay, which is a sea-port. It appears to me, besides, that the circumstance of their being so common in your district, which abounds so much in brackish water, renders the idea probable that the vitriolic and muriatic salts, which are found in wells and in the sea, favour the propagation of these worms, although I have seen them in places where the purest spring water only is drunk.

*Note by Dr. De Carro.*

The Guinea worm not being a disease to which Europeans are subject, the remedies recommended by Mr. Dubois can only be used in the case of worms in the intestinal canal, where the ordinary remedies have not succeeded; and even the employment of assafœtida as a vermifuge is no novelty. But I invite agriculturists to try whether the above remedies may not be applied to sheep, and such animals as are subject to worms in various parts of their bodies.

